# The pgip family in soybean and three other legume species: evidence for a birth-and-death model of evolution

**DOI:** 10.1186/s12870-014-0189-3

**Published:** 2014-07-18

**Authors:** Raviraj M Kalunke, Alberto Cenci, Chiara Volpi, Donal M O’Sullivan, Luca Sella, Francesco Favaron, Felice Cervone, Giulia De Lorenzo, Renato D’Ovidio

**Affiliations:** 1 Dipartimento di Scienze e tecnologie per l’Agricoltura, le Foreste, la Natura e l’Energia, (DAFNE), Università della Tuscia, Via S. Camillo de Lellis snc, Viterbo, Italy; 2 Bioversity International, Commodity systems & genetic resources programme, Parc Scientifique Agropolis II, 1990 Boulevard de la Lironde, Montpellier Cedex 5, 34397, France; 3 NIAB, Huntingdon Road, Cambridge CB3 0LE, UK; 4 Dipartimento Territorio e Sistemi agro-forestali (TESAF), Università di Padova, Agripolis, Viale dell’Università 16, Legnaro (PD), 35020, Italy; 5 Dipartimento di Biologia e Biotecnologie “Charles Darwin”, Sapienza Università di Roma, Piazzale Aldo Moro, 5, Roma, 00185, Italy; 6 Present address: Enza Zaden Italia Research SRL, S.S. Aurelia km 96.710, Tarquinia (VT), 01016, Italy; 7 Present address: School of Agriculture, Policy and Development, University of Reading, Whiteknights, Reading RG6 6AR, UK

**Keywords:** ᅟ

## Abstract

**Background:**

Polygalacturonase-inhibiting proteins (PGIPs) are leucine-rich repeat (LRR) plant cell wall glycoproteins involved in plant immunity. They are typically encoded by gene families with a small number of gene copies whose evolutionary origin has been poorly investigated. Here we report the complete characterization of the full complement of the *pgip* family in soybean (*Glycine max* [L.] Merr.) and the characterization of the genomic region surrounding the *pgip* family in four legume species.

**Results:**

BAC clone and genome sequence analyses showed that the soybean genome contains two *pgip* loci. Each locus is composed of three clustered genes that are induced following infection with the fungal pathogen *Sclerotinia sclerotiorum* (Lib.) de Bary, and remnant sequences of *pgip* genes. The analyzed homeologous soybean genomic regions (about 126 Kb) that include the *pgip* loci are strongly conserved and this conservation extends also to the genomes of the legume species *Phaseolus vulgaris* L., *Medicago truncatula* Gaertn. and *Cicer arietinum* L., each containing a single *pgip* locus. Maximum likelihood-based gene trees suggest that the genes within the *pgip* clusters have independently undergone tandem duplication in each species.

**Conclusions:**

The paleopolyploid soybean genome contains two *pgip* loci comprised in large and highly conserved duplicated regions, which are also conserved in bean, *M. truncatula* and *C. arietinum*. The genomic features of these legume *pgip* families suggest that the forces driving the evolution of *pgip* genes follow the birth-and-death model, similar to that proposed for the evolution of resistance (R) genes of NBS-LRR-type.

## Background

The plant cell wall represents one of the main obstacles to the colonization of the plant tissue by microbial pathogens. To surmount this barrier, most fungal pathogens produce cell wall degrading enzymes (CWDEs), among which endo-polygalacturonases (PGs; EC 3.2.1.15) are secreted at very early stages of the infection process [[1]]. PGs cleave the α-(1-4) linkages between D-galacturonic acid residues in homogalacturonan, causing cell separation and maceration of host tissue. To counteract the activity of PGs, plants possess cell wall glycoproteins, called polygalacturonase-inhibiting proteins (PGIPs), the importance of which in defence has been demonstrated in different plant species [[2]-[12]].

Like the products of many resistance genes, PGIPs belong to the subclass of proteins containing leucine-rich repeats (LRRs) of the extracytoplasmic type (LxxLxLxxNxLT/SGxIPxxLxxLxx) [[13]]. The LRR domain of PGIP is typically formed by 10 imperfect LRRs of 24 residues each and is responsible for the molecular interaction with PGs. The LRRs are organized to form two β-sheets, one of which (sheet B1) occupies the concave inner side of the molecule and contains residues crucial for PG recognition [[14]].

To counteract the many PGs produced by fungal pathogens, plants have evolved a variety of PGIPs with different specificities. Variability is present also within each plant species, since PGIPs are encoded by gene families, comprising 2 members in *Arabidopsis thaliana* [[3]] up to 16 in *Brassica napus* [[15]]. A clear example of intra-specific variation in the inhibition properties against fungal and insect PGs has been reported for members of the bean (*Phaseolus vulgaris* L.) *pgip* family [[16]]. Variation among different family members extends also to the regulation of their expression [[17]].

The soybean (*Glycine max* [L.] Merr.) *pgip* family is composed by at least four genes forming two clusters, one containing *Gmpgip1* and *Gmpgip2*, separated by about 3 kbp, and the other containing *Gmpgip3* and *Gmpgip4*, separated by a maximum distance of 60 Kbp [[18]]. These findings are particularly interesting because soybean is a well-established paleopolyploid plant species. Consequently, the characterization of the full complement of the soybean *pgip* family could provide important information about the forces driving the evolution of this gene family.

In this study, we have characterized the complete set of *pgip* genes in the soybean genotype Williams 82 and demonstrated the existence of two *pgip* loci. The study was extended to other legume species by characterizing a region of about 140 Kb, comprising the single *pgip* locus of bean, and the *pgip* regions in the recently released genomes of *Medicago truncatula* Gaertn. [[19]] and *Cicer arietinum* L. [[20]]. Comparative analysis between the *pgip* regions of these species suggests that the legume *pgip* family follows the birth-and-death model of evolution.

## Results

### Characterization of soybean BAC clones and isolation of two novel *Gmpgip* genes

Seven BAC clones, previously isolated from a soybean BAC library using a *pgip* probe, were analysed with primers specific for *Gmpgip1*, *Gmpgip2*, *Gmpgip3* and *Gmpgip4* [[18]]. None of the BAC clones contained all four *pgip* sequences together. Three of them (95O4, 85 M15, 28B18) contained both *Gmpgip1* and *Gmpgip2* and two (26I2, 6 F5) contained both *Gmpgip3* and *Gmpgip4*. The remaining two BAC clones (1 F11, 62 K14) did not produce a clear amplicon with none of the Gmpgip primer combinations; therefore, they were not analyzed further. The size of the insert contained in each BAC clone, determined by pulsed-field gel electrophoresis (PFGE) following *Not*I digestion, varied between about 50 Kb and 190 Kb (Additional files 1 and 2). Fingerprinting of the BAC clones following *Hind*III digestion showed overlapping profiles for those containing *Gmpgip1* and *Gmpgip2* (95O4, 85 M15, 28B18), and those containing *Gmpgip3* and *Gmpgip4* (26I2, 6 F5) (Additional file 1). BAC end sequencing and shotgun subcloning and sequencing identified two novel *Gmpgip* genes, one in the clone 85 M15 (*Gmpgip5*), the other in the clone 26I2 (*Gmpgip7*). *Gmpgip5* was at the terminal end of 85 M15 and was partial; its complete sequence was obtained by PCR performed on genomic DNA. The coding regions of *Gmpgip5* and *Gmpgip7* contain uninterrupted open reading frames (ORFs) of 1008 and 1011 bp, respectively, including the stop codon. The predicted amino acid sequence of these two ORFs showed the typical PGIP structure, comprising a 21 amino acid signal peptide for secretion in the apoplast, 10 leucine rich repeats (LRRs) of about 24 amino acids each and eight cysteine residues, four each at N- and C-terminal part of the protein (Additional file 3).

Since fingerprint analysis indicated the lack of overlapping portions between the two sets of BAC clones, we hypothesized the existence of two *pgip* loci, one including *Gmpgip1*, *Gmpgip2* and *Gmpgip5*, as identified in the BAC clone 85 M15 and the other one including *Gmpgip3*, *Gmpgip4* and *Gmpgip7*, as identified in the BAC clone 26I2. The recent availability of the soybean genome sequence [[21]] allowed us to confirm the existence of two *pgip* loci and to define the spatial distribution of the *Gmpgip* genes (see below).

### Transcript accumulation and *in vitro* inhibition assays of *Gmpgip5* and *Gmpgip7*

We have previously reported variation in the expression pattern of *Gmpgip* genes (*Gmpgip1*, *Gmpgip2*, *Gmpgip3* and *Gmpgip4*) following infection of soybean hypocotyls with the necrotrophic fungal pathogen *Sclerotinia sclerotiorum* [[18]]. In this work, we extended the study to *Gmpgip5* and *Gmpgip7*. qRT-PCR analysis showed that *Gmpgip5* and *Gmpip7* are expressed in soybean hypocotyls and are induced following the infection with *S. sclerotiorum*. The basal transcript levels of both *Gmpgip5* and *Gmpgip7* are much lower than that of *Gmpgip3*, used as control because it is the most highly expressed *Gmpgip* gene in soybean hypocotyls [[18]]; moreover, basal expression of *Gmpgip7* was higher than that of *Gmpgip5* (Table 1). Expression of *Gmpgip5* decreases during the first 24 hour post infection (hpi) with *S. sclerotiorum*, to greatly increase by more than 1000 fold at 48 hpi, the last time point analyzed, when the tissue is almost completely macerated (Table 2). Upon infection, expression of *Gmpgip7* shows a moderate increase during the first 24 hpi to reach high levels at 48 hpi (Table 2). *Gmpgip3* showed different kinetics of transcript accumulation, with a maximum of five fold increase at 24 hpi and no further increase at 48 h (Table 2).

**Table 1:** **Basal expression of** ***Gmpgip5*** **and** ***Gmpgip7*** **compared with** ***Gmpgip3*** **in soybean hypocotyls prior pathogen infection**

**Genes**	**Ct values**^**a**^
*Gmpgip3*	19.83 ± 1.00
*Gmpgip5*	32.68 ± 0.94
*Gmpgip7*	24.65 ± 0.88
*GmELF1A*	17.13 ± 1.01

^a^Transcript levels were determined by quantitative RT-PCR. *Gmpgip* and *GmELF1A* (housekeeping) genes showed similar amplification efficiencies. *Gmpgip3* crossed the detection of threshold 6 and 13 cycles before *Gmpgip7* and *Gmpgip5* transcripts, respectively. Means of Ct values are based on three technical replicates each of two biological replicates.

**Table 2:** **Time-course expression analysis**^**a**^**of** ***Gmpgip*** **genes in etiolated soybean hypocotyls infected with** ***S*.** ***sclerotiorum***

**Hours post infection (hpi)**	**Relative expression**^**b**^		
	***Gmpgip3***	***Gmpgip5***	***Gmpgip7***
8	1.16 ± 0.55	1.45 ± 1.95	4.38 ± 1.30
16	2.30 ± 0.891	0.56 ± 0.22	5.78 ± 0.17
24	5.76 ± 2.02	0.29 ± 0.32	6.98 ± 5.06
48	5.89 ± 2.69	454.0 ± 34.1	265.5 ± 32.01

^a^Analysis was performed by qRT-PCR. *Gmpgip* and housekeeping genes showed similar amplification efficiencies. Amplicon sizes: *Gmpgip3* (211 bp); *Gmpgip5* (194 bp); *Gmpgip7* (200 bp); *GmELF1A* (195 bp).

^b^Quantification of gene expression was performed using the comparative Ct method (Livak and Schmittgen, 2001). Relative expression of each gene is reported as the fold increase of the transcript level in infected sample relative to each corresponding non-infected control sample and normalized with *GmELF1A*.

Database searches confirmed the presence of expressed sequence tags (ESTs) corresponding to *Gmpgip5* and *Gmpgip7* and showed variation of their expression during development, with *Gmpgip5* ESTs present in hypocotyls and pods and *Gmpgip7* ESTs present in roots and stem (Additional file 4). ESTs for the remaining *Gmpgip* genes were also identified, with *Gmpgip3* being the most represented in soybean tissues (Additional file 4). In particular, about twice more transcripts have been found in the hypocotyl for this gene compared to *Gmpgip5*, confirming the higher level of expression of *Gmpgip3* shown by the qRT-PCR results (Table 1).

In order to verify the inhibition activities of *Gmpgip5* and *Gmpgip7* against fungal PGs, we have expressed these genes in *Nicotiana benthamiana* using a vector based on potato virus X (PVX; [[22]]). Western blot analyses on total protein extracts using an antibody raised against the bean PGIP showed the accumulation of GmPGIP7 and GmPGIP3, which was used as positive control (Additional file 5). On the contrary, no immunodecoration signal was detected in extracts prepared from control plants (non-inoculated or inoculated with the empty vector) (Additional file 5) or from plants infected with the PVX-Gmpgip5 construct (data not shown). Despite considerable effort, all PVX-based attempts to express GmPGIP5 failed.

Total protein extracts of *N. benthamiana* leaves infected with the PVX-Gmpgip7 were then used to test the inhibitory activity of GmPGIP7 against the fungal PGs of *S. sclerotiorum*, *Fusarium graminearum*, *Colletotrichum acutatum* and *Aspergillus niger* (data not shown)*.* No inhibition was observed; on the contrary, all the PGs examined were inhibited by GmPGIP3, used as control (Additional file 5).

### The soybean *pgip* genes are organized in two distinct loci

**Figure 1:**
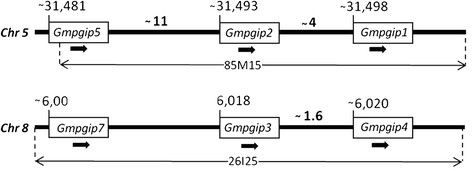
**Genomic organization of the** ***pgip*** **gene family in soybean cv. Williams 82.** Schematic representation of the arrangement of the *Gmpgip* genes in the two loci of the soybean genome. Regions covered by the BAC clones 85 M15 and 26I2 on chromosomes 5 and 8, respectively, are indicated. On the basis of the soybean genome database, 85 M15 starts and ends at 31,481,533 bp and 31,579,062 bp, respectively, whereas 26I2 starts and ends at 5,980,885 and 6,108,916, respectively. Numbers between the genes represent distances in kb as determined on the soybean genome database. Arrows indicate the direction of the coding region from ATG to stop codon. Boxes are not in scale. Chr, chromosome.

Sequence comparison of the *Gmpgip* genes contained within the BAC clones and the assembled soybean genome sequence allowed us to confirm the presence of two *pgip* loci and to determine the arrangement of the *Gmpgip* genes within each of them. Each cluster of *pgip* genes spans a region of similar length (about 18 Kb) on chromosome 5 (in order, *Gmpgip5*, *Gmpgip2* and *Gmpgip1*) and chromosome 8 (*Gmpgip7*, *Gmpgip3* and *Gmpgip4*). All genes are transcribed in the same direction (Figure 1). In addition to these transcribed *pgip* genes, two and one remnant sequences were found on chromosome 5 and 8, respectively. One of the remnants in the locus on chromosome 5 [Gmpgip*(1)] is heavily fragmented, whereas the other two, on chromosomes 5 [Gmpgip*(2)] and 8 (Gmpgip*) encode 65 residues of the C-terminal region and 220 residues of the N- terminal and middle regions, respectively (Additional file 6).

**Figure 2:**
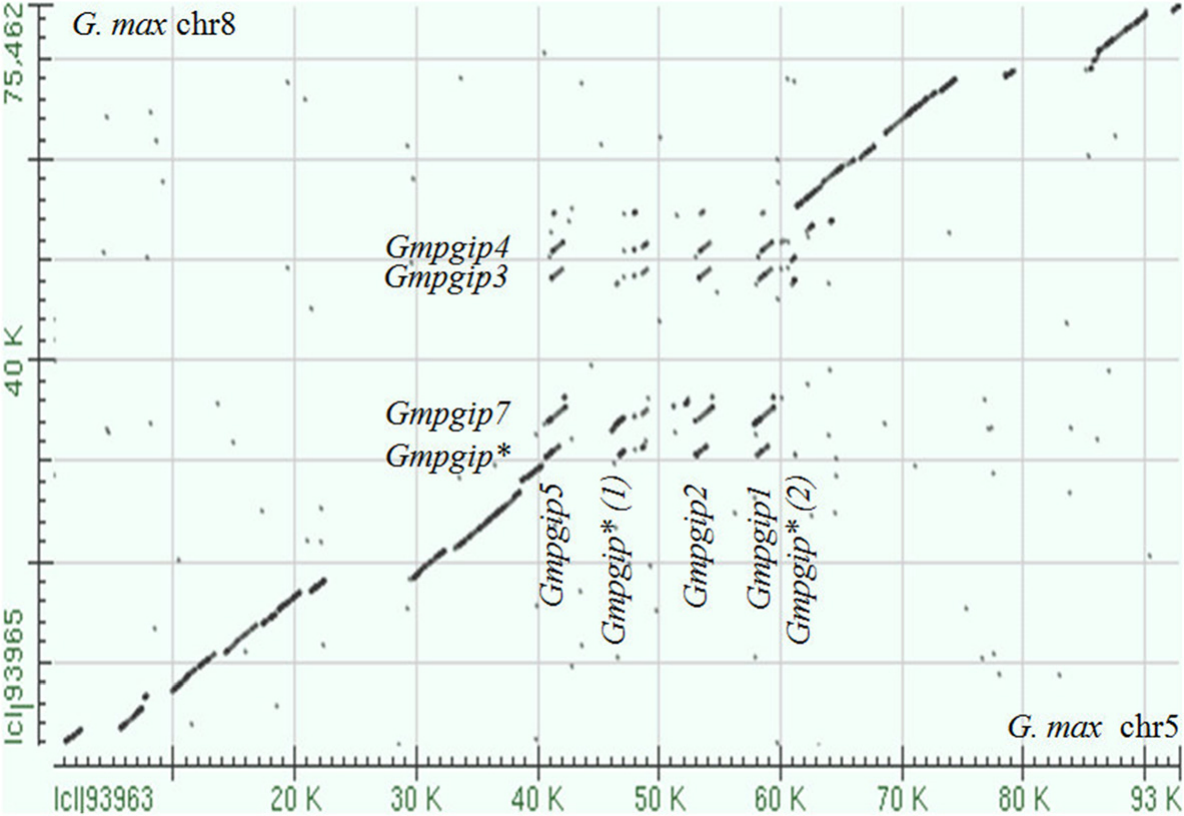
**Bl2seq alignment analysis of the genomic regions encompassing the two** ***pgip*** **loci of** ***G. max*.** Bl2seq analysis was performed between the *pgip* region (_˜_96 Kb) in chromosome 5 and that (_˜_75 Kb) in chromosome 8. Regions flanking the *pgip* loci are collinear in gene order, orientation as well as nucleotide sequence. *, remnant; Chr, Chromosome.

Nucleotide sequences of regions the two soybean *pgip* loci were compared by a Bl2seq analysis, showing that the regions flanking the *pgip* loci are well conserved in nucleotide sequence and collinear in gene order and orientation (Figure 2). The main exception to this collinearity is represented by two major gaps in the alignment, due to LTR retrotransposon insertions in the locus on chromosome 5 (Figure 2). Notably, the region containing the *Gmpgip* copies shows the most exceptions to the sequence collinearity, as no clear diagonal is visible in this region and alignments are limited to the coding regions of different *pgip* members (Figure 2).

In the comparison between loci, only *Gmpgip1* and *Gmpgip3,* on the one side, and *Gmpgip2* and *Gmpgip7,* on the other side*,* share similarity in the 3′ regions, limited to the proximal 200 bp sequences. The 5′ regions of the different *Gmpgip* genes are also strongly divergent and sequence divergence in these regions is reflected also in the composition of *cis*-elements. Sequence analysis. limited to known *cis*-acting elements regulating genes involved in the defence response, showed that all six *Gmpgip* genes contain sequences sharing identity with these elements; differing, however, in types and numbers. For example *Gmpgip3* contains the highest number of W-box elements, whereas it lacks sequences matching BIHD1OS2 elements (Additional file 7).

### Structural analysis of the bean *pgip* locus

For the characterization of the bean *pgip* locus, the bean BAC clones 129 F4 and 10G1 spanning the bean *pgip* locus were isolated from a genomic library prepared from BAT93 genotype. The 129 F4 and 10G1 clones contain an insert of about 37150 bp and 107473 bp, respectively, with an overlapping segment of 5201 bp [[16]]. These clones were completely sequenced allowing the characterization of a total of 139420 bp, with the *pgip* region spanning about 50 Kb. This region represents the only *pgip* locus present in the bean genome and contains four intronless *Pvpgip* genes (*Pvpgip1*Bat, *Pvpgip2*Bat, *Pvpgip3*Bat and *Pvpgip4*Bat) transcribed in the same direction [[16]].

The assembled BAC sequence of *P. vulgaris* BAT93 was mapped in two different genomic regions of the recently available genome sequence of *P. vulgaris* accession G19833. A major portion of the assembled BAT93 sequence (1..126624) was mapped on chromosome 2 [complement (36019507..36152120)] and shown to contain several annotated genes (from Phvul.002G200800.1 to Phvul.002G201900.1), whereas the remaining 13 Kb were mapped on chromosome 1 (49570303..49583535) and did not contain annotated genes. Three hypotheses can be made to explain this discrepancy: 1) the BAC 10G1 that contains the two regions is chimeric, i.e. two independent portions of BAT93 genome were cloned in the same BAC; 2) an assembly error, involving the analyzed regions, is present in the whole genome sequence, and 3) a translocation took place that differentiated the *P. vulgaris* BAT3 and G19833 accessions. Since the shorter region accounts only for about 10% of the entire BAC assembled sequence and shows a potentially different origin from the main BAC sequence assembly, we excluded it from subsequent analyses.

In order to analyze the structure of the region containing the bean *pgip* genes, a Blast2seq analysis (sequence aligned with itself), limited to the first 62 Kb of the assembled sequence that contain the four bean *pgip* genes, was performed. In addition to the diagonal alignment, several short alignments are present (Additional file 8). These include two retrotransposable elements (positions around 10 Kb and 40 Kb), which showed off-diagonal alignment of their Long Tandem Repeats (LTRs), as tipically shown by LTR retrotransposon ends (Additional file 8). The four *pgip* genes align to each other, and alignment extending beyond the coding regions was observed only between *PvBpgip1* and *PvBpgip2* and between *PvBpgip3* and *PvBpgip4* (Additional file 8). Conversely, the alignment between *pgip* pairs *PvBpgip1*/*PvBpgip3*, *PvBpgip2*/*PvBpgip3*, *PvBpgip1*/*PvBpgip4*, and *PvBpgip2*/*PvBpgip4* is limited to the coding regions.

### Comparison of *pgip* loci in four Fabaceae species

The assembled bean sequence (1..126624) containing the cluster of *pgip* genes was compared with the soybean genome and with the recent genome sequence releases of *M. truncatula* [[19]] and *C. arietinum* [[20]]. Like in bean, the genomes of *M. truncatula* and *C. arietinum* contain only one *pgip* locus. The *pgip* locus of *M. truncatula* spans a region of about 25 Kb containing a cluster of two genes with uninterrupted open reading frames (MTR_119s0023, *Mtpgip1*; MTR_119s0021, *Mtpgip2*), one possible pseudogene, annotated with an intron of 40 bp to restore the correct open reading frame (MTR_119s0017, *Mtpgip3*), and one remnant corresponding to a sequence encoding a C-terminal 53 residue PGIP fragment. The products encoded by *Mtpgip1 Mtpgip2* and *Mtpgip3* are shown in Additional file 9. They all contain a signal peptide for secretion to the apoplast, the typical 10 LRRs and eight cysteine residues, four each in N- and C-terminal portion of the protein; MtPGIP1 contains an additional cysteine in the C-terminal region (Additional file 9). Similarly, the *pgip* locus of *C. arietinum* spans a region of about 30 K and contains two *pgip* genes (Additional file 10), one of which (LOC101504619, *Capgip2*) is interrupted by a fragment of about 17 Kb in the middle of the coding region. The putative protein encoded by *Capgip1* (LOC101505245) also contains the typical PGIP features described above (Additional file 10). Taken together, these sequence analyses highlight that the typical PGIP structure is strongly conserved among and within all these *pgip* families. This conservation is accompanied, as expected, by the typical variation within LRRs composing each protein, and LRRs of different proteins.

**Figure 3:**
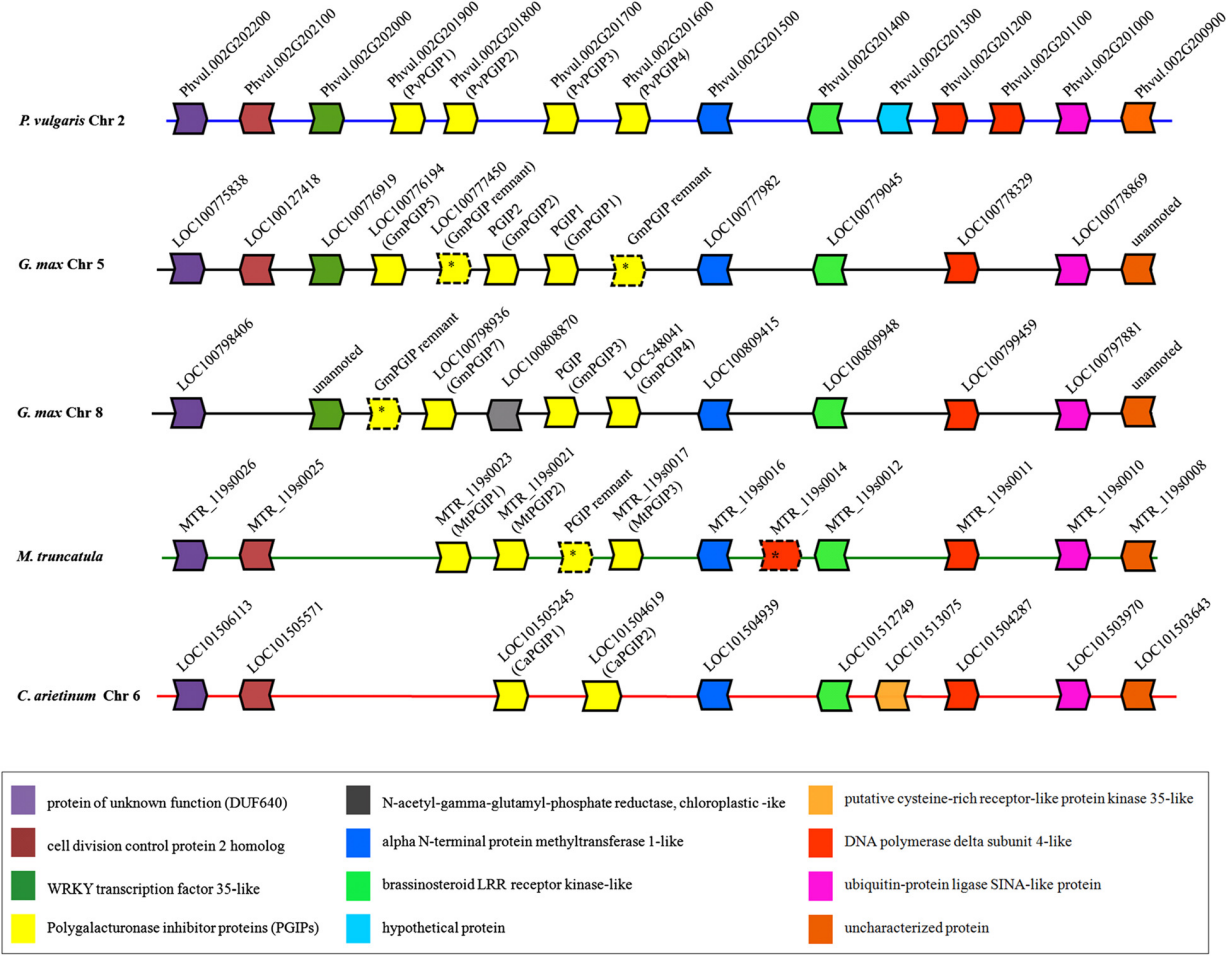
**Schematic representation of genomic regions containing the** ***pgip*** **loci in** ***Fabaceae*** **species.** Genomic regions containing the *pgip* loci were analysed for their shared synteny. Each block-arrow represents a predicted gene and the direction of the coding region from ATG to stop codon. Colored block-arrows are genes with a homolog. The gray block-arrow indicates a gene with no homolog. Genomic position from the left to the right element: *P. vulgaris* chromosome 2 (36,203,534 to 36,041,993 bp), *G. max* chromosome 5 (31,445,987 to 31,542,227 bp), *G. max* chromosome 8 (59,75,212 to 60,52,779 bp), *M. truncatula* (unplaced genomic scaffold 119) and *C. aretinum* (14,308,467, complement to 14,227,083 bp). *, remnant; Chr, chromosome.

Sequence comparison between the flanking regions of the *pgip* clusters in all four species, showed few genes and a very well conserved order, with few exceptions. Of the ten bean genes flanking the *pgip* cluster, only Pv_202000 and Pv_201300 are not conserved in all four legume species (Figure 3). Moreover, Pv_201200 and Pv_201100 exist as duplicated genes only in the bean genome, and Pv 202100 was lost in soybean chromosome 8 (Figure 3).

**Figure 4:**
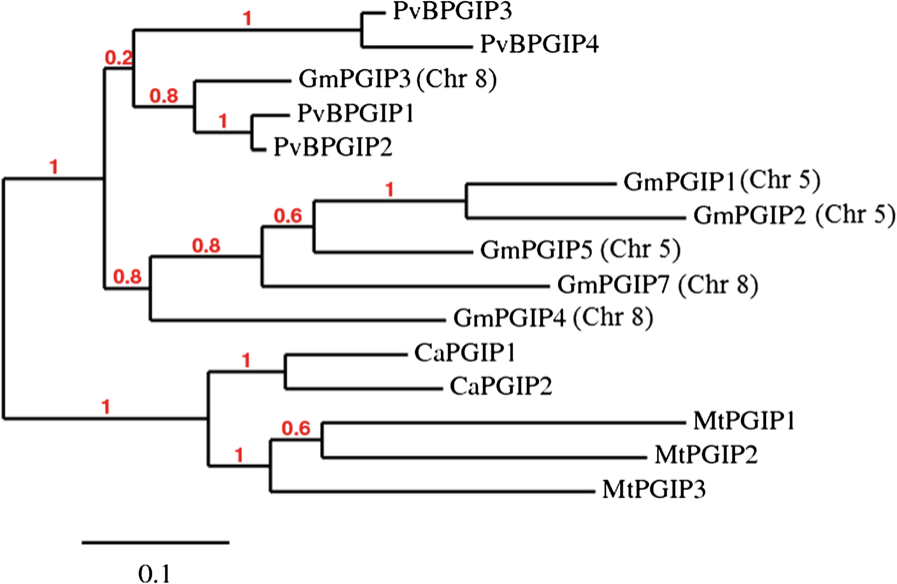
**Phylogenetic tree showing the relationship among different PGIPs from** ***Fabaceae*** **species.** The unrooted tree was constructed by a Maximum likelihood approach (PhyML) using the deduced amino acid sequences. Gm, *Glycine max*; PvB, *Phaseolus vulgaris*; Mt, *Medicago truncatula; Ca, Cicer aretinum;* Chr, Chromosome.

PGIP protein sequences from these four Fabaceae species (*P. vulgaris*, *G. max*, *M. truncatula*, and *C. arietinum*) were aligned by MUSCLE and a phylogenetic tree was constructed by a Maximum likelihood approach (PhyML). As shown in Figure 4, the unrooted tree revealed that: i) *M. truncatula* and *C. arietinum pgip* copies are in species-specific clusters, suggesting that copy amplification took place after the divergence of species; ii) the *M. truncatula* and *C. arietinum pgip* clusters are close to each other and form a very well supported cluster; iii) the *G. max* and *P. vulgaris pgip* members are in a different cluster, consistent with the general taxonomic relationships of these members of the *Fabaceae*. Furthermore, *G. max* and *P. vulgaris* genes are distributed in three main clusters: a) a cluster including PvBPGIP1, PvBPGIP2 and GmPGIP3; b) a cluster including PvBPGIP3 and PvBPGIP4 and c) a cluster containing the remaining *G. max* genes.

## Discussion

In this work, we have demonstrated that the full complement of the soybean *pgip* family is composed of six transcribed genes located in two different loci in the sub-terminal and terminal regions of chromosomes 5 and 8, respectively. Each *pgip* locus contains both complete and disrupted coding regions indicating that a pseudogenization (“death”) process is active in the family. As assumed for NBS-LRR *R*-genes [[23]], the clusters of recently duplicated *pgip* copies should provide a reservoir of genetic variation from which novel *pgip* genes can evolve. The resemblance to NBS-LRR *R*-genes is further supported by previous findings showing that variation between *pgip* genes of different species or copies within a gene family is mainly due to single substitutions within the LRR domain and in particular in the xxLxLxx solvent-exposed region [[15],[16],[18],[24]]. Like in *R*-genes, models of codon evolution suggest the presence, in the solvent-exposed region of PGIP, of sites under positive selection [[25]-[27]], and functional analysis demonstrated that single substitutions or a short deletion within this region can cause changes in the inhibition properties of PGIP against fungal PGs [[16],[28]-[33]].

An interesting feature of the soybean *pgip* loci, which are included in a larger duplicated region, is the high sequence conservation of the regions surrounding the *pgip* clusters that contrasts with the variability in the intergenic regions between *pgip* genes. Differences in the regions external to the *pgip* gene clusters are limited to small indels and to the presence of two transposable elements only in the region of chromosome 8. This conserved organization is typical of the paleopolyploid soybean genome, which underwent two rounds of Whole Genome Duplication (WGD) [[34],[35]]. Recent analysis of the complete soybean genome sequence has revealed that indeed this is composed to a large extent by blocks of duplicated genes [[21]]; however. before the availability of the complete assembled soybean genome, data had indicated that the soybean genome was a mosaic of alternating homeologous regions retaining high sequence conservation and regions showing very low conservation [[35]].

In the homeologous regions containing the *pgip* clusters, the striking conservation is interrupted only by the remarkable sequence variability in the intergenic regions between the *pgip* genes. This low sequence conservation includes the proximal 5′ flanking regions, suggesting a differential regulation of the different *pgip* genes. Indeed, the six *pgip* genes show variation in *cis*-acting elements known to regulate defense response genes, and their expression patterns following pathogen infection show clear differences. The two novel *pgip* genes, *Gmpgip5* and *Gmpgip7*, are poorly expressed in soybean hypocotyls but are strongly induced at late stages of infection with the fungal pathogen *S. sclerotiorum.* This expression pattern is similar to that of *Gmpgip2* and differs from that of *Gmpgip1, Gmpgip3* and *Gmpgip4*, which all show a more prompt induction following *S. sclerotiorum* infection [[18]]. Diversification of gene regulation following fungal pathogen infections or stress stimuli has been reported for other *pgip* gene families, including those of *Arabidopsis* [[3]], bean [[16]] and *B. napus* [[15]], and suggests adaptation against stresses. However, this possibility in soybean is still poorly supported at the protein level, since, at present, inhibition activity against fungal PGs has been shown *in vitro* only for the product encoded by *Gmpgip3*, which is also the most expressed soybean family member [[18]]. In fact, although GmPGIP1, GmPGIP2 and GmPGIP4 [[18]] and now also GmPGIP7 were expressed in *N. benthamiana* using PVX as a vector, they did not show any inhibitory activity. The lack of *in vitro* inhibition activity of GmPGIPs does not exclude the possibility that they can inhibit PGs from different sources not yet examined, or that only the *in planta* environment provides a suitable context to support the interaction with PGs. This last possibility has been suggested by Joubert et al. [[6]] who found a reduction of symptoms caused by the activity of *Botrytis cinerea* BcPG2 on plant tissue when co-infiltrated with *Vitis vinifera* VvPGIP1, although no interaction between VvPGIP1 and BcPG2 was detected *in vitro*. Moreover, as suggested previously [[18]], the lack of inhibition activity by GmPGIP1, GmPGIP2 GmPGIP4 and now GmPGIP7 towards fungal PGs may reflect a different physiological role *in planta*. This possibility is supported by several observations in different plant species. For example, OsFOR1, a rice protein possessing PG inhibiting capabilities, affects the formation and/or maintenance of floral organ primordia in rice [[36]]; levels of PGIP1 determine the timing of radicle protrusion in Arabidopsis [[37]], and *Vvpgip1* can affect gene expression and cell wall structure in transgenic tobacco plants [[38],[39]].

The genomic regions encompassing the *pgip* genes of soybean were also compared to the corresponding region of bean, which shares a very close phylogenetic relationship with soybean. The *pgip* gene family of the bean genotype BAT93, which comprises four clustered paralogs, has been previously characterized [[16]]. We have now extended the analysis to the bean BAT93 sequences flanking the *pgip* cluster and found a very strong conservation in the distribution of the genes compared to duplicated soybean regions encompassing the *pgip* loci. Of the ten bean genes flanking the *pgip* cluster, seven were conserved in both soybean chromosomes 5 and 8. Pv_202100 was lost only on soybean chromosome 8, and the duplicated bean genes Pv_201200 and Pv_201100 exist as a single copy in both soybean chromosome 5 and 8.

Conservation of the genomic *pgip* region is also evident in the more distantly related legume species *M. truncatula* and *C. arietinum*, whose assembled genomes have been recently released [[19],[20]]. Both species possess a single *pgip* locus, with a cluster organization of the paralogs, and regions flanking the *pgip* array that maintain the strongly conserved distribution of the genes observed in the soybean and bean. Of the ten bean genes that surround the *pgip* cluster, seven are conserved in all four legume species. The finding that most of the *pgip* genes are organized in species-specific phylogenetic clusters indicates that the *pgip* copies within each cluster were independently formed after speciation. An exception to this observation is represented by GmPGIP3, PvPGIP1 and PvPGIP2. In fact, the soybean and bean PGIPs form separated clusters in maximum likelihood-based gene trees and, within the bean cluster, the four PvPGIPs form two well separated groups. However, as previously highlighted [[16],[18]], the soybean GmPGIP3 groups with PvPGIP1 and PvPGIP2, suggesting that the duplication originating the ancestors of PvPGIP1/PvPGIP2 and PvPGIP3/PvPGIP4 took place before the separation of *Glycine* and *Phaseolus* lineages. In this context, it is noteworthy that the high sequence conservation of PvPGIP2 extends across a range of *P. vulgaris* germplasm and other *Phaseolus* species, suggesting an adaptive significance [[30]].

## Conclusions

The paleopolyploid soybean genome contains two *pgip* loci comprised in large and highly conserved duplicated regions, likely originating from WGD. The region encompassing the *pgip* locus is also conserved in bean, *M. truncatula* and *C. arietinum*. The genomic features of these legume *pgip* families, which include inferred recent duplications and pseudogenization of *pgip* copies, suggest that the forces driving the evolution of *pgip* genes follow the birth-and-death model, similarly to that proposed for the evolution of NBS-LRR-type *R* genes [[23]].

## Methods

### Plant material and infection experiments

Soybean seeds (*G. max* [L.] Merr. cv. Williams 82) were germinated by placing them on previously sterilized and soaked paper towels which were then rolled and incubated for five days in the dark at 24°C.

The B-24 isolate of *S. sclerotiorum* (Lib. De Bary) was grown for three days at 24°C on potato dextrose agar to obtain mycelium for the inoculation of soybean seedlings.

Infection experiments were performed by inoculating the middle region of etiolated soybean hypocotyls with actively growing mycelium of *S. sclerotiorum* as described by Favaron et al. [[40]]. Soybean seedlings were placed horizontally on plastic trays. Roots were covered with a layer of moist paper towel. Plants were inoculated by placing small plugs (5 × 2 mm) of mycelium-colonized agar, cut from marginal zones of actively growing colonies, along the middle region of hypocotyls. Control soybean seedlings were mock inoculated with sterile agar medium. After incubated at 24°C in the dark hypocotyl segments (approximately 5 mm) cut transversally with a razor blade exactly below the agar plugs were collected at 0, 8, 16, 24, and 48 h after inoculation, frozen in liquid nitrogen and stored at –80°C for subsequent analyses. Lesion of the tissue increased during time and at 48 hpi it affected most part of the hypocotyls as reported by Favaron et al. [[40]]. Two independent infection experiments were performed.

### RNA extraction and RT-PCR analysis

Total RNA was extracted using RNeasy Plant Mini Kit (Qiagen, Italy) according to manufacturer’s instructions. RNA concentration was determined both spectrophotometrically and by densitometric analysis of rRNA fragment following agarose gel electrophoresis. QuantiTect® Reverse Transcription Kit (Qiagen) was used to remove genomic DNA contamination and to synthesize cDNA. Elimination of genomic DNA from cDNA preparation was verified by PCR with primers aligned in different exons for gene Translation elongation factor (*GmELF1A*) and Glucose-6-phosphate dehydrogenase (*GmG6PD*) as described by Miranda et al. [[41]].

The quantitative real-time PCR experiments were performed using the iCycler (Bio-Rad, Italy) and using master mix iQTMSYBER Green Supermix (BioRad, Italy), containing the SYBR Green I DNA binding dye. Each reaction was made in triplicate. Primers were designed using Primer 3 software (http://fokker.wi.mit.edu/primer3/, [[42]]) on the basis of the *Gmpgip* genes and have the following sequences (sense and antisense, respectively): GmPGIP3-3 F 5′-ACCCCAACCCTAATCGGTCA-3′ and GmPGIP3-3R 5′-AGGTGATTCCGACGAGATTG-3′ for *Gmpgip3*; GmPGIP5-1 F 5′-ACCGGACTCCTTCGGCTACTTCC-3′ and GmPGIP5-1R 5′- TGTTTCCCAGATACATGTGCC-3′ for *Gmpgip5*; GmPGIP7-1 F 5′- TAAGGGTGTCAAAGACCTTGTT-3′and GmPGIP7-1R 5′- CACTTGTTATGAGCGTACAGC-3′ for *Gmpgip7*; GmELF1A-F 5′-GACCTTCTTCGTTTCTCGCA-3′ and GmELF1A-R 5′-GAACCTCTCAATCACACGC-3′ for *GmELF1A* [[41]]. Total reaction volume was 20 μl and included 10 μl (2×) master mix, 100 ng of cDNA, 0.5 μl (10 μM) of each forward and reverse primers and volume was adjusted with water. The PCR reaction conditions were: one cycle at 50°C for 2 min, 94°C for 15 min, then 40 cycles at 95°C for 15 sec, 60°C for 50 sec and 72°C for 50 sec. Primer specificity was confirmed by nucleotide sequencing (MWG, Germany) of amplicon. The Ct values of target genes (*Gmpgip3, Gmpgip5* and *Gmpgip7*) and reference gene (*GmELF1A*) were used for further relative expression analysis by using the 2^-ΔΔ^CT method [[43]]. Relative induction level was relative to the corresponding non infected sample at each time point analyzed. Calculation and statistical analyses were performed by Gene Expression Macro™ Version 1.1 (Bio-Rad, Italy). The qRT-PCR experiments included three replicas for each sample in two different biological replicas. PCR efficiency (ε) was calculated for each gene from the slope of linear-regression of the threshold cycle versus log dilution serial of the cDNA according to equation ε = (10^(-1/slope)-1)*100.

### PVX-mediated expression of GmPGIP5 and GmPGIP7, and immunoblotting

The coding region of *Gmpgip5* and *Gmpgip7* was amplified by PCR with primers including restriction sites for *Cla*I and *Sal*I or *Nru*I at the 5′ and 3′ ends, respectively. The amplified fragments were double digested with *Cla*I and *Sal*I or *Nru*I and cloned into corresponding sites of the pPVX201 expression vector. The plasmids obtained were used to inoculate *N. benthamiana* plants using 30 μg of plasmid DNA per plant as described by Baulcombe et al. [[22]]. Transiently expressed GmPGIP7 was extracted from leaves of *N. benthamiana* plants infected with single PVX-Pgip constructs or with the empty vector. Leaves were homogenized in 1 M NaCl (2 ml/g), incubated with gentle shaking for 1 h at 4°C, and centrifuged 20 min at 10,000 g. Supernatant was filtered through Miracloth (Calchem, USA), centrifuged to remove debris and stored at -20°C. Protein concentration was determined with the Coomassie Plus™ (Bradford) assay kit (Pierce, Rockford, IL, USA). SDS-PAGE and immunoblotting were performed as previously described [[18]]. Polyclonal antibodies raised against bean PGIP were used for immunoblotting experiments.

### Fungal growth, PG preparation and enzymatic assays

Fungal growth and PG preparation were performed as previously described for *A. niger* [[44]], *S. sclerotiorum* isolate B-24 [[45]], and *C. acutatum* isolate SHK788 [[16]]. *F. graminearum* isolate 3827 [[46]]. Inhibitory activity of PGIP was performed as previously described by D’Ovidio et al. [[18]]. PG activity was expressed as reducing units (RU). One RU was defined as the amount of enzyme required to release reducing groups at 1 μmol min^-1^ using D-galacturonic acid as standard.

### Screening of genomic libraries and sequencing

The Bacterial artificial chromosome (BAC) library (prepared from *G. max* cv. Williams 82) was purchased from the Clemson University Genomics Institute (CUGI; Clemson, SC, USA). Screening and sequencing of BAC clone was performed as previously described by D’Ovidio et al. [[18]]. The bean BAC clones 129 F4 and 10G1 spanning the bean *pgip* locus were isolated from 16.603 recombinant clones of a genomic library prepared from BAT93 genotype [[16]].

Sequencing reactions were performed using the “ABI PRISM dye terminator cycle sequencing ready reaction” kit and DNA sequences were determined with the semiautomatic ABI PRISM 310 sequencer (Applied Biosystem, Monza, Italy). Sequences were also determined through the MWG-BIOTECH AG (Ebersberg, Germany) and PRIMM Srl. Sequencing Services (Milano, Italy).

### Nucleic acid manipulation and amino sequence analysis

Nucleic acid manipulation, PCR, and cloning were performed according to the standard procedures [[47]]. DNAMAN software (Lynnon, BioSoft, Quebec, Canada) was used for nucleotide and amino acid sequence analyses. Signal peptide for GmPGIP5 and GmPGIP7 was predicted using http://wolfpsort.org/ [[48]]. The 5′ flanking region of *Gmpgip* genes was scanned for presence of the *cis*-elements using PLACE (http://www.dna.affrc.go.jp/PLACE/signalscan.html), a database of plant *Cis*-acting regulatory DNA elements [[49]].

### Phylogenetic analysis

BLASTp analysis was performed by using bean PGIP sequences to identify the already annotated PGIP genes and detect the occurrence of partial PGIP sequences (remnants) in *G. max*, *M. truncatula* and *C. arietinum* genomes.

The PGIP protein sequences from the four Fabaceae species (*P. vulgaris*, *G. max*, *M. truncatula*, and *C. arietinum*) were aligned by MUltiple Sequence Comparison by Log- Expectation (MUSCLE) and used for reconstructing phylogenetic tree. This was performed on web interface www.phylogeny.fr [[50]], using the PhyML software based on the Maximum likelihood principle.

### Comparative analysis

The assembled sequences of the BAC clones 129 F4 and 10G1 (accession number HG964426) from the *P. vulgaris* genotype BAT93 were mapped in the *P. vulgaris* genome sequence by Bl2seq using the www.phytozome.org web interface.

Protein sequences of the genes spanning from Phvul.002G200900.1 to Phvul.002G202200.1 on the bean genome sequence were used as query in Bl2seq to detect most similar sequences in the sequenced genomes of *G. max*, *C. arietinum* and *M. truncatula*.

## Competing interests

The authors declare that they have no competing interests.

## Authors’ contributions

RMK carried out the molecular analyses and contributed to the first draft of the paper; AC performed sequence and phylogenetic analyses; CV performed BAC library screening, clones characterization and heterologous expression analyses; DOS, supervised BAC library screening and clones characterization; LS performed infection experiments; FF, FC and GDL critically discussed the data and paper; RD designed the research, coordinated it and wrote the paper. All authors read, edited and approved the final manuscript.

## Additional files

## Supplementary Material

Additional file 1: **Insert size estimation of soybean BAC clones and fingerprinting analysis.** A) The size of soybean BAC clones was determined by pulsed-field gel electrophoresis (PFGE) following the *Not*I digestion. 1, 26I2; 2, 95O4; 3, 6 F5; 4, 28B18; 5, 85 M15. M1 and M2 indicate the PFGE molecular mass ladder and Lambda-DNA/*Hind*III ladder, respectively. B) Fingerprinting analysis. BAC clones were digested with *Hind*III and separated using 1.0% agarose gel. 1, 6 F5; 2, 28B18; 3, 95O4; 4, 26I2; 5, 85 M15; M, 1 kb DNA ladder. Click here for file

Additional file 2: **BAC clones isolated by screening a BAC library of *G. max*** **cv. Williams 82 using a soybean** ***pgip*** **probe.** Insert size was determined by pulsed-field gel electrophoresis (PFGE) following *Not*I digestion. Click here for file

Additional file 3: **Alignment of the deduced amino acid sequences of** ***G. max* PGIPs.** Numbering is referred to the GmPGIP1 sequence and starts from the first residue of the mature protein. Regions A–D were predicted according to crystallographic analysis of the bean PvPGIP2 (Di Matteo et al. 2003, Proceedings of the National Academy of Sciences, 100, 10124-10128). The xxLxLxx region is boxed. Empty spaces have been added to better show identity/similarity among LRR sequences within a single protein. The predicted signal peptide region (region A) was determined using Wolfpsort (http://wolfpsort.org/; Horton et al. 2007, Nucleic Acids Research (Web Server issue), 35: W585–W587). Dots represent identical amino acid residues; dashes indicate missing amino acids. Cysteine residues are underlined. *Gmpgip1, Gmpgip2* and *Gmpgip5* are in the *pgip* locus on chromosome 5. *Gmpgip3*, *Gmpgip4* and *Gmpgip7* are on chromosome 8. Click here for file

Additional file 4: **Gene expression patterns of the soybean *pgip*** **genes as inferred from expressed sequence tags (ESTs) counts found in public databases**^**a**^**.** Click here for file

Additional file 5: **Western blot of total protein extract from *N. benthamiana*** **plants inoculated with PVX-pgip constructs and agarose diffusion assay for PGIP inhibition.** A) Western blot analysis was performed using total protein extract from *N. benthamiana* plants inoculated with individual PVX 201-based constructs for the expression of GmPGIP3 or GmPGIP7 or the empty vector, as a control. 1, protein ladder; 2, PVX 201 (empty vector); 3, GmPGIP7 (5 μg); 4, GmPGIP7 (10 μg); 5, GmPGIP7 (20 μg); 6, GmPGIP3 (10 μg). B) Agarose diffusion assay using crude protein extract from *N. benthamiana* plants inoculated with the PVX-Gmpgip3 or PVX-Gmpgip7 constructs or the empty vector, as a control. The assay was performed using 0.011 reducing units of *S. sclerotiorum* endopolygalacturonase (SsPG). The absence of halo indicates the inhibition of PG activity. 1, SsPG; 2, SsPG plus GmPGIP3 (1 μg); 3, SsPG plus boiled GmPGIP (1 μg); 4, SsPG plus GmPGIP7 (20 μg); 5, SsPG plus boiled GmPGIP7 (20 μg); 6, SsPG plus empty PVX 201 vector (20 μg); 7, SsPG plus boiled empty PVX 201 vector (20 μg). Similar results were obtained with the PG of *F. graminearum*, *C. acutatum* and *A. niger.* GmPGIP3 inhibited to completion all four PGs, whereas GmPGIP7 did not show any inhibition activity (data not shown). Click here for file

Additional file 6: **Alignment of the deduced amino acid sequences of remnant *G. max* PGIPs.** GmPGIP3 was used as reference gene for sequence alignment. Numbering is referred to the GmPGIP3 sequence and starts from the first residue of the mature protein. Regions A–D were predicted according to crystallographic analysis of the bean PvPGIP2 (Di Matteo et al. 2003, Proceedings of the National Academy of Sciences, 100, 10124-10128). The xxLxLxx region is boxed. The predicted signal peptide region (region A) was determined using Wolfpsort (http://wolfpsort.org/; Horton et al. 2007, Nucleic Acids Research (Web Server issue), 35: W585–W587). The remnants GmPGIP* (1), which is heavily fragmented, and GmPGIP* (2) are located on chromosome 5. The reconstructed GmPGIP* (1) protein sequence exhibits a putative signal peptide for secretion (region A) and a 299-amino acid mature protein. GmPGIP*(2) corresponds to a 65-amino acid C-terminal fragment. The remnant GmPGIP*, located on chromosomes 8, correspond to a PGIP fragment comprising the putative signal peptide and a 220-amino acid portion of the mature protein. Dots indicate identical amino acids; dashes indicate missing amino acids. Empty spaces have been added to better show identity/similarity among LRR sequences within a single protein. Cysteine residues are underlined. *, remnant; Chr, Chromosome. Click here for file

Additional file 7: ***Cis*-acting regulatory DNA elements related to pathogen-induced expression.** The 5′ flanking region sequence (~1 Kb) of each *Gmpgip* gene was analysed using PLACE database (http://www.dna.affrc.go.jp/PLACE/). Click here for file

Additional file 8: **Blast2seq analysis of the region containing the bean *pgip*** **genes.** A nucleotide sequence limited to 62 Kb containing the *pgip* genes (*PvBpgip1*, *PvBpgip2*, *PvBpgip3*, and *PvBpgip4*) was self-aligned. A red rectangular box represents the Long Tandem Repeats (LTR) retrotransposons. A blue rectangular box represents the specific *pgip* genes. Ellipses indicate alignments among conserved regions around *Pgip* genes (blue) and between the two LTR retroelements (red). Click here for file

Additional file 9: **Alignment of the deduced amino acid sequences from *M. truncatula*** **PGIPs.** Numbering is referred to the MtPGIP1 sequence and starts from the first residue of the mature protein. Regions A–D were predicated according to crystallographic analysis of the bean PvPGIP2 (Di Matteo et al. 2003, Proceedings of the National Academy of Sciences, 100, 10124-10128). The xxLxLxx region is boxed. Predicted signal peptide region (region A) was determined using Wolfpsort (http://wolfpsort.org/; Horton et al. 2007, Nucleic Acids Research (Web Server issue), 35: W585–W587). Empty spaces have been added to better show identity/similarity among LRR sequences within a single protein. Dots represent identical amino acid residues; dashes indicate missing amino acids. Cysteine residues are underlined. Click here for file

Additional file 10: **Alignment of the deduced amino acid sequences from *C. arietinum*** **PGIPs.** CaPGIP1 sequence is numbered starting from the first residue of the mature protein. Regions A–D were predicated according to crystallographic analysis of the bean PvPGIP2 (Di Matteo et al. 2003, Proceedings of the National Academy of Sciences, 100, 10124-10128). The xxLxLxx region is boxed. Empty spaces indicate gaps to maximize identity/similarity between sequences. Predicted signal peptide region (region A) was determined using Wolfpsort (http://wolfpsort.org/; Horton et al. 2007, Nucleic Acids Research (Web Server issue), 35: W585–W587). Empty spaces have been added to better show identity/similarity among LRR sequences within a single protein. Dots represent identical amino acid residues; dashes indicate missing amino acids. Cysteine residues are underlined. Click here for file
