# Supplementary material for: The pgip family in soybean and three other legume species: evidence for a birth-and-death model of evolution
Source: BMC Plant Biol. 2014 Jul 18;14:189. doi: 10.1186/s12870-014-0189-3 (PMC4115169; doi:10.1186/s12870-014-0189-3)
Supplement: Additional file 1: — Insert size estimation of soybean BAC clones and fingerprinting analysis. A) The size of soybean BAC clones was determined by pulsed-field gel electrophoresis (PFGE) following the NotI digestion. 1, 26I2; 2, 95O4; 3, 6 F5; 4, 28B18; 5, 85 M15. M1 and M2 indicate the PFGE molecular mass ladder and Lambda-DNA/HindIII ladder, respectively. B) Fingerprinting analysis. BAC clones were digested with HindIII and separated using 1.0% agarose gel. 1, 6 F5; 2, 28B18; 3, 95O4; 4, 26I2; 5, 85 M15; M, 1 kb DNA ladder. [file s12870-014-0189-3-S1.doc]

**Additional file 1.** Insert size estimation of soybean BAC clones and fingerprinting analysis. **A**) The size of soybean BAC clones was determined by pulsed-field gel electrophoresis (PFGE) following the *Not*I digestion. 1, 26I2; 2, 95O4; 3, 6F5; 4, 28B18; 5, 85M15. M1 and M2 indicate the PFGE molecular mass ladder and Lambda-DNA/*Hind*III ladder, respectively. **B**) Fingerprinting analysis. BAC clones were digested with *Hind*III and separated using 1.0% agarose gel. 1, 6F5; 2, 28B18; 3, 95O4; 4, 26I2; 5, 85M15; M, 1 kb DNA ladder.
