# Supplementary material for: The pgip family in soybean and three other legume species: evidence for a birth-and-death model of evolution
Source: BMC Plant Biol. 2014 Jul 18;14:189. doi: 10.1186/s12870-014-0189-3 (PMC4115169; doi:10.1186/s12870-014-0189-3)
Supplement: Additional file 2: — BAC clones isolated by screening a BAC library of G. max cv. Williams 82 using a soybean pgip probe. Insert size was determined by pulsed-field gel electrophoresis (PFGE) following NotI digestion. [file s12870-014-0189-3-S2.docx]

**Additional file 2.** BAC clones isolated by screening a BAC library of *G. max* cv. Williams 82 using a soybean *pgip* probe. Insert size was determined by pulsed-field gel electrophoresis (PFGE) following *Not*I digestion.

| **BAC clones** | **Insert size (K)** |
| --- | --- |
| 95O4 | 190 |
| 85M15 | 100 |
| 28B18 | 150 |
| 26I2 | 130 |
| 6F5 | 50 |
