# Supplementary material for: The pgip family in soybean and three other legume species: evidence for a birth-and-death model of evolution
Source: BMC Plant Biol. 2014 Jul 18;14:189. doi: 10.1186/s12870-014-0189-3 (PMC4115169; doi:10.1186/s12870-014-0189-3)
Supplement: Additional file 3: — Alignment of the deduced amino acid sequences of G. max PGIPs. Numbering is referred to the GmPGIP1 sequence and starts from the first residue of the mature protein. Regions A–D were predicted according to crystallographic analysis of the bean PvPGIP2 (Di Matteo et al. 2003, Proceedings of the National Academy of Sciences, 100, 10124-10128). The xxLxLxx region is boxed. Empty spaces have been added to better show identity/similarity among LRR sequences within a single protein. The predicted signal peptide region (region A) was determined using Wolfpsort (http://wolfpsort.org/; Horton et al. 2007, Nucleic Acids Research (Web Server issue), 35: W585–W587). Dots represent identical amino acid residues; dashes indicate missing amino acids. Cysteine residues are underlined. Gmpgip1, Gmpgip2 and Gmpgip5 are in the pgip locus on chromosome 5. Gmpgip3, Gmpgip4 and Gmpgip7 are on chromosome 8. [file s12870-014-0189-3-S3.docx]

**Region A**

GmPGIP1 MSLLSIL--LLIVMSF SL ALS

GmPGIP2 ..H....FL..VAL.. TP ...

GmPGIP5 ..R....I.....L.. .S ...

GmPGIP3 ..K....F-..V L.. .S V..

GmPGIP4 .--IMV.--.IV LYL .AP...

GmPGIP7 ..HS.T.FLF...LF. TP ...

**Region B**

GmPGIP1 ELCNPQDKQTLLQIKKELGNPTTLSSWHPKTDCCNNS WVGVSCDTVT PTYRV**^52^**

GmPGIP2 .....L...A.....R......N....N........N .......... .....

GmPGIP5 .....R...V..K..............L.T...... .......... Q....

GmPGIP3 .........A......D..........LLT.....RT .L......D. Q....

GmPGIP4 K... ....V............K....L.T..Y.D TI.E..A.V.DSNNQ.C**..**

GmPGIP7 ........EA.......F.........L.TS.....N ......ANK. QS...

**Region C** xxLxLxx

GmPGIP1 DNLDLSELN LRKPYP IPPS VGSLPCL**^78^**

GmPGIP2 .......IY .T.... .... I.N..Y.

GmPGIP5 H.....D.. .P...S ..F. I.NI.Y.

GmPGIP3 ND....D.. .P...S .... IAN..Y.

GmPGIP4 .I.Y..H.. .P.... .... I.N..Y.

GmPGIP7 NH...ND.. .P.... .... ..N..Y.

GmPGIP1 KFLYITNNPNIVGT IPTT ITKLTKL**^103^**

GmPGIP2 ...F...S...... .... .......

GmPGIP5 E..S..GT...I.. ..P. .......

GmPGIP3 N..S.SRT.TLI.Q ..SA .A...Q.

GmPGIP4 NY..LIDT .FF.A ..SS .AN..N.

GmPGIP7  N..S...TN.L... ..P. .....M.

**Additional file 3.** Alignment of the deduced amino acid PGIP sequences from *Glycine max*.

Numbering is referred to the GmPGIP1 sequence and starts from the first residue of the mature protein. Regions A–D were predicted according to crystallographic analysis of the bean PvPGIP2 (Di Matteo et al. 2003, Proceedings of the National Academy of Sciences, 100, 10124-10128). The xxLxLxx region is boxed. Empty spaces have been added to better show identity/similarity among LRR sequences within a single protein. The predicted signal peptide region (region A) was determined using Wolfpsort (http://wolfpsort.org/; Horton et al. 2007, Nucleic Acids Research (Web Server issue), 35: W585–W587). Dots represent identical amino acid residues; dashes indicate missing amino acids. Cysteine residues are underlined. *Gmpgip1, Gmpgip2* and *Gmpgip5* are in the *pgip* locus on chromosome 5. *Gmpgip3*, *Gmpgip4* and *Gmpgip7* are on chromosome 8.

GmPGIP1 RELNIRYTN ISGQ IPHF LSQIKAL**^127^**

GmPGIP2 ...Y....S V... .... ...M.T.

GmPGIP5 .N.Y.K... V... ..R. .....T.

GmPGIP3 .Y.Y.TH.. V..P ..D. ...I.T.

GmPGIP4 NY...T... V..T ..D. ..H..T.

GmPGIP7  ...Q..F.. V..E .... .....T.

GmPGIP1 GFLDLSNNK LSGN LP SWLPSLPDL**^151^**

GmPGIP2 Q..EF..C**.** .... .. T........

GmPGIP5  E.....Y.. .... ..A ......N.

GmPGIP3 VT..F.Y.T ...K ..A. .S...N.

GmPGIP4 VSI.F.Y.N .... ..A. .S...N.

GmPGIP7 ESIIFHY.N F... ..P ......N.

GmPGIP1 YGISFDNNY ISGP IPDLFAS VSERF**^176^**

GmPGIP2 ..VA....R ...A ...S.GF ..K..

GmPGIP5 V.....G.R ...A ...S.GY FPKS.

GmPGIP3 V..T..G.Q ...A ...SYG. F.KL.

GmPGIP4 GEMI.TG.R ...A ...S.G. F..EL

GmPGIP7 .RV.L.G.R ...T ...S.G. F.DSL

GmPGIP1 GFISLSGNR LIGK IPAS LGKPDM**^199^**

GmPGIP2 .YMT..... .S.. ..S. ...L.L

GmPGIP5 VML...R.. .T.. ...T .A.L.V

GmPGIP3 TSMTI.R.. .T.. ...T FANLNL

GmPGIP4 ILMR..R.. .T.. ...T .A.LNL

GmPGIP7 KLMTF.D.. .T.E ...T .A.L.F

GmPGIP1 KIVDLSRNM LEGDASVLFGSEKHT**^223^**

GmPGIP2 .T......K .............R.

GmPGIP5 .F.Y..K.. ......L........

GmPGIP3 AF....... ..........T..N.

GmPGIP4 RFL...... .............D.

GmPGIP7 DF....Q.. ...............

GmPGIP1 ERIYLANNLFAFDLGKVRLSKT L**^246^**

GmPGIP2 .M....H......F....VP.V .

GmPGIP5 RHM..G..S.......LG.... .

GmPGIP3 QK.L..K.ML.......G...N .

GmPGIP4 VQ.N.GK.NL.......EF.EI .

GmPGIP7 LQ.N....S....F....V... .

GmPGIP1 GLLDVGHNL IYGT LPKGLTS LKDL**^270^**

GmPGIP2 DS......R L... ....... ..N.

GmPGIP5 EG..LS..R L... ....... ....

GmPGIP3 NG..LRN.R .... ..Q...A ..F.

GmPGIP4 AI..LR..R .... ..Q...A ..H.

GmPGIP7 LI.YLS..R L..A ..E.... ..N.

GmPGIP1 YYLDVSYNN LCGE IPRGGK**^289^**

GmPGIP2 .RF.....K .... ......

GmPGIP5 ......... ...K ......

GmPGIP3 HSFN..F.D .... ..Q..N

GmPGIP4 TK.N..N.. .... ..Q..N

GmPGIP7 GTF.....E ...K .....R

**Region D**

GmPGIP1 LQEFDASLYANNKCLCGSPLPSCKRF**^315^**

GmPGIP2 ...I.E.F..................

GmPGIP5 .......T..H...............

GmPGIP3 M.R..V.S..DD.........P.T

GmPGIP4 ..RIKVNS..H..........A.TLVN

GmPGIP7 ..KI.V.S.SH..........K..HL
