# Supplementary material for: The pgip family in soybean and three other legume species: evidence for a birth-and-death model of evolution
Source: BMC Plant Biol. 2014 Jul 18;14:189. doi: 10.1186/s12870-014-0189-3 (PMC4115169; doi:10.1186/s12870-014-0189-3)
Supplement: Additional file 4: — Gene expression patterns of the soybean pgip genes as inferred from expressed sequence tags (ESTs) counts found in public databases a . [file s12870-014-0189-3-S4.docx]

**Additional file 4.** Gene expression patterns of the soybean *pgip* genes as inferred from expressed sequence tags (ESTs) counts found in public databases^a^.

^a^ EST profiles show approximate gene expression patterns, according to NCBI database and the cDNA library sources (as reported by sequence submitters).

*Breakdown by body sites, with numbers indicating transcripts per million (TPM) and reflecting gene activity.
