# Supplementary material for: The pgip family in soybean and three other legume species: evidence for a birth-and-death model of evolution
Source: BMC Plant Biol. 2014 Jul 18;14:189. doi: 10.1186/s12870-014-0189-3 (PMC4115169; doi:10.1186/s12870-014-0189-3)
Supplement: Additional file 5: — Western blot of total protein extract from N. benthamiana plants inoculated with PVX-pgip constructs and agarose diffusion assay for PGIP inhibition. A) Western blot analysis was performed using total protein extract from N. benthamiana plants inoculated with individual PVX 201-based constructs for the expression of GmPGIP3 or GmPGIP7 or the empty vector, as a control. 1, protein ladder; 2, PVX 201 (empty vector); 3, GmPGIP7 (5 μg); 4, GmPGIP7 (10 μg); 5, GmPGIP7 (20 μg); 6, GmPGIP3 (10 μg). B) Agarose diffusion assay using crude protein extract from N. benthamiana plants inoculated with the PVX-Gmpgip3 or PVX-Gmpgip7 constructs or the empty vector, as a control. The assay was performed using 0.011 reducing units of S. sclerotiorum endopolygalacturonase (SsPG). The absence of halo indicates the inhibition of PG activity. 1, SsPG; 2, SsPG plus GmPGIP3 (1 μg); 3, SsPG plus boiled GmPGIP (1 μg); 4, SsPG plus GmPGIP7 (20 μg); 5, SsPG plus boiled GmPGIP7 (20 μg); 6, SsPG plus empty PVX 201 vector (20 μg); 7, SsPG plus boiled empty PVX 201 vector (20 μg). Similar results were obtained with the PG of F. graminearum, C. acutatum and A. niger. GmPGIP3 inhibited to completion all four PGs, whereas GmPGIP7 did not show any inhibition activity (data not shown). [file s12870-014-0189-3-S5.docx]

**Additional file 5.** Western blot of total protein extract from *N*. *benthamiana* plants inoculated with PVX-pgip constructs and agarose diffusion assay for PGIP inhibition.

**A**) Western blot analysis was performed using total protein extract from *N*. *benthamiana* plants inoculated with individual PVX 201-based constructs for the expression of GmPGIP3 or GmPGIP7 or the empty vector, as a control. 1, protein ladder; 2, PVX 201 (empty vector); 3, GmPGIP7 (5 µg); 4, GmPGIP7 (10 µg); 5, GmPGIP7 (20 µg); 6, GmPGIP3 (10 µg). **B**) Agarose diffusion assay using crude protein extract from *N*. *benthamiana* plants inoculated with the PVX-Gmpgip3 or PVX-Gmpgip7 constructs or the empty vector, as a control. The assay was performed using 0.011 reducing units of *S. sclerotiorum* endopolygalacturonase (SsPG). The absence of halo indicates the inhibition of PG activity. 1, SsPG; 2, SsPG plus GmPGIP3 (1 μg); 3, SsPG plus boiled GmPGIP (1 μg); 4, SsPG plus GmPGIP7 (20 μg); 5, SsPG plus boiled GmPGIP7 (20 μg); 6, SsPG plus empty PVX 201 vector (20 μg); 7, SsPG plus boiled empty PVX 201 vector (20 μg). Similar results were obtained with the PG of *F. graminearum*, *C. acutatum* and *A. niger.* GmPGIP3 inhibited to completion all four PGs, whereas GmPGIP7 did not show any inhibition activity (data not shown).
