# Supplementary material for: The pgip family in soybean and three other legume species: evidence for a birth-and-death model of evolution
Source: BMC Plant Biol. 2014 Jul 18;14:189. doi: 10.1186/s12870-014-0189-3 (PMC4115169; doi:10.1186/s12870-014-0189-3)
Supplement: Additional file 6: — Alignment of the deduced amino acid sequences of remnant G. max PGIPs. GmPGIP3 was used as reference gene for sequence alignment. Numbering is referred to the GmPGIP3 sequence and starts from the first residue of the mature protein. Regions A–D were predicted according to crystallographic analysis of the bean PvPGIP2 (Di Matteo et al. 2003, Proceedings of the National Academy of Sciences, 100, 10124-10128). The xxLxLxx region is boxed. The predicted signal peptide region (region A) was determined using Wolfpsort (http://wolfpsort.org/; Horton et al. 2007, Nucleic Acids Research (Web Server issue), 35: W585–W587). The remnants GmPGIP* (1), which is heavily fragmented, and GmPGIP* (2) are located on chromosome 5. The reconstructed GmPGIP* (1) protein sequence exhibits a putative signal peptide for secretion (region A) and a 299-amino acid mature protein. GmPGIP*(2) corresponds to a 65-amino acid C-terminal fragment. The remnant GmPGIP*, located on chromosomes 8, correspond to a PGIP fragment comprising the putative signal peptide and a 220-amino acid portion of the mature protein. Dots indicate identical amino acids; dashes indicate missing amino acids. Empty spaces have been added to better show identity/similarity among LRR sequences within a single protein. Cysteine residues are underlined. *, remnant; Chr, Chromosome. [file s12870-014-0189-3-S6.doc]

**Additional file 6.** Alignment of the deduced amino acid sequences of PGIP remnants from *Glycine max*.

GmPGIP3 was used as reference gene for sequence alignment. Numbering is referred to the GmPGIP3 sequence and starts from the first residue of the mature protein. Regions A–D were predicted according to crystallographic analysis of the bean PvPGIP2 (Di Matteo et al. 2003, Proceedings of the National Academy of Sciences, 100, 10124-10128). The xxLxLxx region is boxed. The predicted signal peptide region (region A) was determined using Wolfpsort (http://wolfpsort.org/; Horton et al. 2007, Nucleic Acids Research (Web Server issue), 35: W585–W587). The remnants *GmPGIP* (1)*, which is heavily fragmented, and *GmPGIP* (2)* are located on chromosome 5. The reconstructed GmPGIP* (1) protein sequence exhibits a putative signal peptide for secretion (region A) and a 299-amino acid mature protein. GmPGIP*(2) corresponds to a 65-amino acid C-terminal fragment. The remnant *GmPGIP**, located on chromosomes 8, correspond to a PGIP fragment comprising the putative signal peptide and a 220-amino acid portion of the mature protein. Dots indicate identical amino acids; dashes indicate missing amino acids. Empty spaces have been added to better show identity/similarity among LRR sequences within a single protein. Cysteine residues are underlined. *, remnant; Chr, Chromosome.
