# Supplementary material for: The pgip family in soybean and three other legume species: evidence for a birth-and-death model of evolution
Source: BMC Plant Biol. 2014 Jul 18;14:189. doi: 10.1186/s12870-014-0189-3 (PMC4115169; doi:10.1186/s12870-014-0189-3)
Supplement: Additional file 7: — Cis -acting regulatory DNA elements related to pathogen-induced expression. The 5′ flanking region sequence (~1 Kb) of each Gmpgip gene was analysed using PLACE database (http://www.dna.affrc.go.jp/PLACE/). [file s12870-014-0189-3-S7.docx]

**Additional file 7.** *Cis*-acting regulatory DNA elements related to pathogen-induced expression. The 5’ flanking region sequence (~1 Kb) of each *Gmpgip* gene was analysed by using PLACE (<http://www.dna.affrc.go.jp/PLACE/>).

| ***Cis* element** | **Sequence** | ***Cis*-element positions^a^** | | | | | |
| --- | --- | --- | --- | --- | --- | --- | --- |
|  |  | ***Gmpgip1*** | ***Gmpgip2*** | ***Gmpgip3*** | ***Gmpgip4*** | ***Gmpgip5*** | ***Gmpgip7*** |
| ASF1MOTIFCAMV^1^ | TGACG |  |  | - 961^a^ | - 177^a^ |  |  |
| BIHD1OS^2^ | TGTCA | - 121^b^  - 234^b^  - 650^b^ | - 242^a^  - 185^b^  - 384^b^ |  | - 421^a^  - 226^b^ | - 856^a^ | - 478^a^  - 426^a^  - 89^b^ |
| WRKY71OS^3^ | TGAC | -733^a^  - 676^a^  - 124^a^  -115^a^  - 549^b^  - 829^b^ | - 614^a^  - 100^a^  - 284^b^ | - 962^a^  - 759^a^  - 556^a^  - 142^a^  - 102^a^  - 368^b^  - 469^b^ | - 149^a^  - 100^a^  - 284^b^ | -934^a^,  -132^a^ | -776^a^  - 612^a^  - 110^a^  - 102^a^  - 58^b^  - 300^b^  - 697^b^ |
| WBOXATNPR1^3^ | TTGAC | - 115^a^ |  | - 962^a^  - 856^a^  - 102^a^  - 368^b^ |  | - 934^a^ | -612^a^  -102^a^  -300^b^ |
| WBOXNTERF3^4^ | TGACY |  |  | - 758^a^  - 857^a^  - 142^a^  - 102^a^  - 367^b^ |  | - 933^a^  - 132^a^ | - 613^a^  - 109^a^  - 102^a^  - 57^b^ |
| GT1GMSCAM4^5^ | GAAAAA | -354^b^ | - 26^b^  - 839^b^  - 802^b^  - 534^b^ | - 818^a^  - 579^a^ |  |  | - 722^a^  - 28^b^ |

^1^‘TGACG’ motif involved in transcriptional activation of several genes by auxin and/or salicylic acid (Redman et al. 2002, Plant Cell Report, 21, 180-185)

^2^‘TGTCA’motif involved in disease resistance responses as reported by Luo et al. (2005, Plant biology, 7, 459-468)

^3^ Minimal W-box elements, involved in pathogen response as reported by Ciolkowski et al., (2008, Plant Molecular Biology, 68, 81-92)

^4^W-box element involved in activation of genes by wounding (Nishiuchi et al. 2004, Journal of Biological Chemistry, 279, 5355-5361)

^5^ "GT-1 motif" involved in induction genes by plant pathogen response (Park et al. 2004, Plant Physiology, 135, 2150-2161)

^a^Position of the *cis* element with respect to the translation start (5’ end/3’ end).

^b^Sequence on the complementary strand.
