# Supplementary material for: The pgip family in soybean and three other legume species: evidence for a birth-and-death model of evolution
Source: BMC Plant Biol. 2014 Jul 18;14:189. doi: 10.1186/s12870-014-0189-3 (PMC4115169; doi:10.1186/s12870-014-0189-3)
Supplement: Additional file 8: — Blast2seq analysis of the region containing the bean pgip genes. A nucleotide sequence limited to 62 Kb containing the pgip genes (PvBpgip1, PvBpgip2, PvBpgip3, and PvBpgip4) was self-aligned. A red rectangular box represents the Long Tandem Repeats (LTR) retrotransposons. A blue rectangular box represents the specific pgip genes. Ellipses indicate alignments among conserved regions around Pgip genes (blue) and between the two LTR retroelements (red). [file s12870-014-0189-3-S8.docx]

**Additional file 8.** Blast2seq analysis of the region containing the bean *pgip* genes.

A nucleotide sequence limited to 62 Kb containing the *pgip* genes (*PvBpgip1*, *PvBpgip2*, *PvBpgip3*, and *PvBpgip4*) was self-aligned. A red rectangular box represents the Long Tandem Repeats (LTR) retrotransposons. A blue rectangular box represents the specific *pgip* genes. Ellipses indicate alignments among conserved regions around *Pgip* genes (blue) and between the two LTR retroelements (red).
