# Supplementary material for: The pgip family in soybean and three other legume species: evidence for a birth-and-death model of evolution
Source: BMC Plant Biol. 2014 Jul 18;14:189. doi: 10.1186/s12870-014-0189-3 (PMC4115169; doi:10.1186/s12870-014-0189-3)
Supplement: Additional file 9: — Alignment of the deduced amino acid sequences from M. truncatula PGIPs. Numbering is referred to the MtPGIP1 sequence and starts from the first residue of the mature protein. Regions A–D were predicated according to crystallographic analysis of the bean PvPGIP2 (Di Matteo et al. 2003, Proceedings of the National Academy of Sciences, 100, 10124-10128). The xxLxLxx region is boxed. Predicted signal peptide region (region A) was determined using Wolfpsort (http://wolfpsort.org/; Horton et al. 2007, Nucleic Acids Research (Web Server issue), 35: W585–W587). Empty spaces have been added to better show identity/similarity among LRR sequences within a single protein. Dots represent identical amino acid residues; dashes indicate missing amino acids. Cysteine residues are underlined. [file s12870-014-0189-3-S9.docx]

**Additional file 9.** Alignment of the deduced amino acid PGIP sequences from *M.* *truncatula*.

Numbering is referred to the MtPGIP1 sequence and starts from the first residue of the mature protein. Regions A–D were predicated according to crystallographic analysis of the bean PvPGIP2 (Di Matteo et al. 2003, Proceedings of the National Academy of Sciences, 100, 10124-10128). The xxLxLxx region is boxed. . Predicted signal peptide region (region A) was determined using Wolfpsort (http://wolfpsort.org/; Horton et al. 2007, Nucleic Acids Research (Web Server issue), 35: W585–W587). Empty spaces have been added to better show identity/similarity among LRR sequences within a single protein. Dots represent identical amino acid residues; dashes indicate missing amino acids. Cysteine residues are underlined.
